# Lifeact-TagGFP2 alters F-actin organization, cellular morphology and biophysical behaviour

**DOI:** 10.1038/s41598-019-40092-w

**Published:** 2019-03-01

**Authors:** Luis R. Flores, Michael C. Keeling, Xiaoli Zhang, Kristina Sliogeryte, Núria Gavara

**Affiliations:** School of Engineering and Materials Science, Queen Mary University of London, Mile End Road, E1 4NS London, UK

**Keywords:** Cellular imaging, Cytoskeleton

## Abstract

Live-imaging techniques are at the forefront of biology research to explore behaviour and function from sub-cellular to whole organism scales. These methods rely on intracellular fluorescent probes to label specific proteins, which are commonly assumed to only introduce artefacts at concentrations far-exceeding routine use. Lifeact, a small peptide with affinity for actin microfilaments has become a gold standard in live cell imaging of the cytoskeleton. Nevertheless, recent reports have raised concerns on Lifeact-associated artefacts at the molecular and whole organism level. We show here that Lifeact induces dose-response artefacts at the cellular level, impacting stress fibre dynamics and actin cytoskeleton architecture. These effects extend to the microtubule and intermediate filament networks as well as the nucleus, and ultimately lead to altered subcellular localization of YAP, reduced cell migration and abnormal mechanical properties. Our results suggest that reduced binding of cofilin to actin filaments may be the underlying cause of the observed Lifeact-induced cellular artefacts.

## Introduction

Live-cell actin visualization is routinely performed and presented in a large percent of cell biology research, including studies where actin or the cytoskeleton may only be secondary players on the observations reported. Lifeact, a small peptide with affinity for actin microfilaments^1–3^, has become one of the gold standards in live cell imaging of actin structures in particular, and overall cell morphology in general. A number of reports have assessed the suitability of Lifeact as a cytoskeletal marker, focusing primarily on qualitative observations of which structures are preferentially labelled by Lifeact relative to other probes such as phalloidin, utrophin or actin-GFP^4,5^. It has been recently reported that Lifeact alters actin filament arrangement and dynamics in fission yeast cells^6^. Similarly, strong *in vivo* Lifeact expression causes sterility in fruit flies^7^, associated with severe actin defects and multiple nuclei in follicle cells. In addition, the detrimental effects of strong Lifeact expression in cells appear to be linked to the specific promoter and fluorescent protein tag used^8,9^. The aforementioned studies have focused on highlighting the abnormal morphologies, dynamics and overall behaviour of cells associated with strong Lifeact expression. Nevertheless, it remains to be discerned whether low to mid-level expression of Lifeact results in unaltered actin dynamics, or conversely if Lifeact induces broad dose-dependent effects on the actin cytoskeleton. Such an understanding is still missing to better define the experimental conditions under which Lifeact is to be considered a suitable probe to image actin structures.

## Results

### Cell cultures transduced with Lifeact-TagGFP2 display altered morphologies

In our experiments, we first performed an overnight transduction of human Mesenchymal Stem Cells (hMSCs) with increasing concentrations (presented as Multiplicity of Infection - MOI) of commercial adenoviral vectors delivering rAVCMV-LifeAct-TagGFP2 plasmid. We transduced cells with MOI ranging from low levels (MOI 100) up to the highest dose recommended by the supplier (MOI 1000). Samples were fixed 1–7 days post transduction, co-stained with TRITC-phalloidin and DAPI, and subsequently imaged via standard epifluorescence microscopy at 20x magnification (Supplementary Fig. 1 and Table 1). When pooling together data at the population level, we found a statistically significant increase in GFP intensity for experiments using higher MOIs (Supplementary Fig. 2). Likewise, we found that GFP levels significantly changed with increasing expression time, with the peak of expression occurring 5 days post transduction. Surprisingly, we found comparable trends when we measured simple parameters that describe cellular morphology and actin assembly, such as cell area and filamentous-actin (F-actin) amount (Supplementary Fig. 3). These analogous temporal and concentration-dependent trends observed at the population level suggested that intracellular Lifeact may result in altered cellular and cytoskeletal morphology.

### Lifeact-TagGFP2 alters actin organization in a dose-response manner

**Figure 1:**
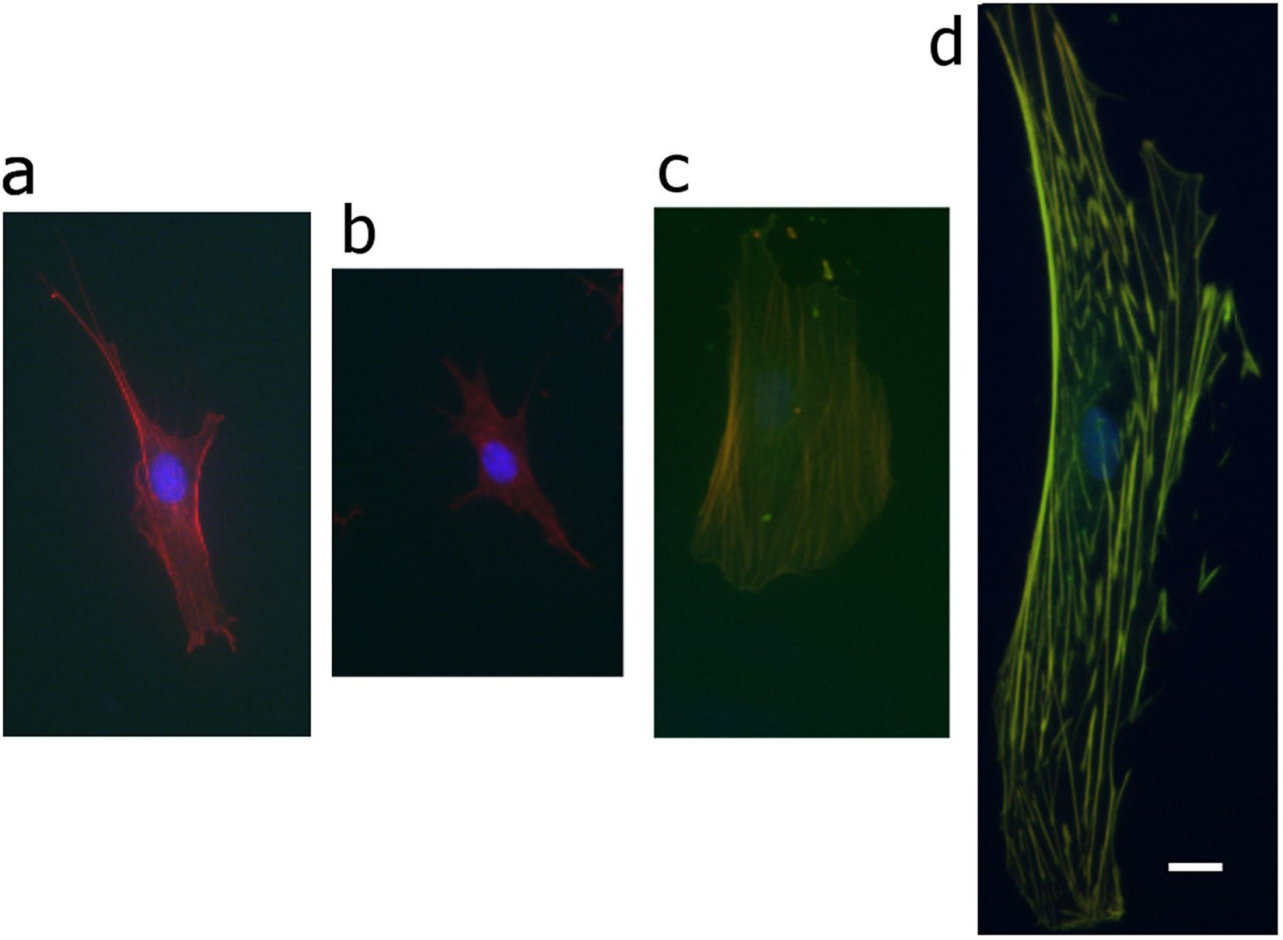
Characteristic phenotypes of cells expressing increasing amounts of Lifeact-TagGFP2 and co-stained with phalloidin-TRITC and DAPI. (**a**) Cell cultured on a coverslip dish that was not transduced, (**b**) cell sorted as ‘no-effect regime’, (**c**) cell sorted as ‘dose-response-regime’, (**d**) cell sorted as ‘saturation plateau’. Scale bar corresponds to 15 µm and is the same for all cells pictured.

Traditional methods based on population averages may mask the fact that a great variation exists in the uptake of plasmid or vector copy number for each cell within a transduced cell culture^10,11^. Thus, to accurately assess the dose-response effects of Lifeact expression at the cellular level, we devised an alternative approach based on pooling together single-cell data according to their measured Lifeact expression, irrespective of initial MOI or time post-transduction. Two critical aspects of our methodological approach need to be emphasised here. First, the quantification of parameters related to cytoskeleton organization and cell morphology was performed using images obtained through TRITC-phalloidin staining, i.e. independently of Lifeact-TagGFP2 driven fluorescence. By doing so the cytoskeleton of cells with low Lifeact-TagGFP2 expression (displaying low GFP fluorescence intensities, Fig. 1b) could be resolved with similar accuracy to those expressing larger Life-GFP levels (Fig. 1d). Second, we took advantage of the 1:1 stoichiometry between the Lifeact peptide and the GFP tag, and measured for each cell its total GFP fluorescence as a surrogate indicator of Lifeact expression^12^. Furthermore, we extended our previously-developed image quantification pipelines^13,14^ to describe in a multiplex fashion the organization of the cytoskeleton and nucleus of individual cells.

**Figure 2:**
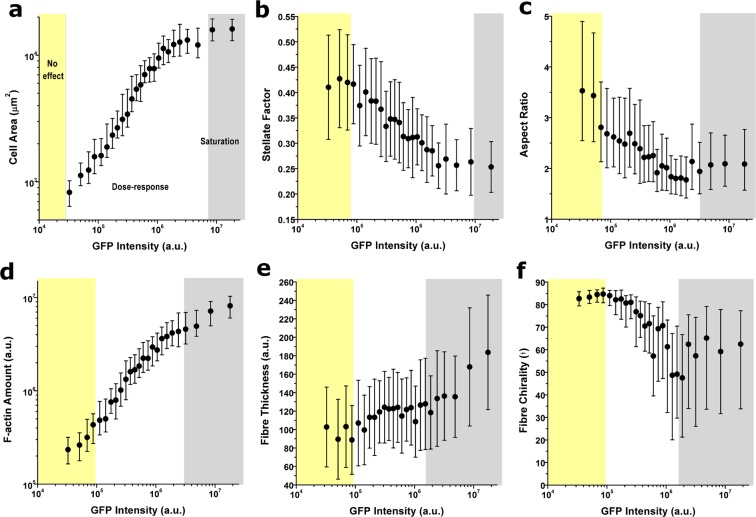
Dose-response curves quantifying the effect of Lifeact expression in cell spread area (**a**), cell perimeter stellate factor (**b**), aspect ratio (**c**), f-actin amount (**d**), fibre thickness (**d**) and chirality of fibres (**f**). Values for >100 cells were pooled together to compute each individual data point. Data is presented as geometric mean (**a**,**d**), mean (**b**,**e**) or median (**c**,**f**) error bars indicate geometric standard deviation, standard deviation or Q1-Q3, accordingly. Background colours indicate the regimes where cells display no Lifeact-induced effect (yellow background), a dose-response trend (white background) and a saturation plateau (gray background), as identified from analyses of peak changes in variability in the neighbourhood of each point for each parameter plotted.

We constructed dose-response curves (DRC) to depict morphometric parameters as a function of intracellular GFP intensity and observed clear morphological trends linking increased Lifeact expression with altered cellular phenotypes (Fig. 2 and Supplementary Fig. 4). In particular, cells displaying the highest Lifeact expression had 10-fold larger spread areas, smaller aspect ratios and a less stellate morphology (Fig. 2). Concurrently, when assessing actin organization, Lifeact expression caused a 50-fold increase in F-actin assembly (Fig. 2d), leading to stress fibres that were longer (Supplementary Fig. 4b), thicker (Fig. 2e) and with an increasing radial orientation (Fig. 2f). To verify that the effects observed were associated with Lifeact rather than its fluorescent tag, we generated similar DRC with cells transduced with the same promoter and a GFP tag only (Supplementary Fig. 5). While the DRCs obtained were not so broad in terms of expression levels reached, we verified that the dose-response behaviour was lost when only GFP was transduced. In particular, multiple comparisons analysis (Supplementary Fig. 5) showed that, even with the highest transduction levels reached with the pCMV-EGFP plasmid there is no significant difference with the lowest dosage with the same treatment. Furthermore, multiple comparisions analysis also shows that, for the majority of parameters, there are significant differences between cells with the highest dose of pCMV-EGFP and cells displaying similar GFP fluorescence levels but transduced with pCMV-Lifeact-Tag2. Additional experiments using Lifeact-TagGFP2 recombinant protein delivered into the cellular cytoplasm using a membrane fusion reagent resulted again in a dose-response behaviour that displayed marked overlap with the results obtained using adenoviral transduction of Lifeact-TagGFP2 (Supplementary Fig. 5). Similar as before, for the majority of parameters, multiple comparisons analysis showed that there were significant differences between cells treated with the highest dose of pCMV-EGFP plasmid and cells treated with the highest doses of recombinant plasmid. Conversely, there were no significant differences between cells treated with the highest dosages of pCMV-Lifeact-Tag2 plasmid versus the recombinant protein.

Of note, DRCs generated for all cytoskeletal parameters had at least two marked regimes (Fig. 2 and Supplementary Fig. 4), namely a dose-response behaviour for low to mid expression levels of Lifeact-TagGFP2 (*white background area in panels*) followed by a saturation plateau at very high expression levels (*gray background area in panels*). In addition, for some cytoskeletal parameters measured we could also identify a range of low Lifeact-TagGFP2 expression levels for which no dose-response effect was observed (*yellow background area in panels*). Similar dose-response trends were also obtained when NIH/3T3 or COS-7 cells were transduced with Lifeact-TagGFP2 vector, even though overall values for parameters such as cell area or F-actin amount were different, as expected for different cell types (Supplementary Fig. 6). Altogether, these data evidences that Lifeact-TagGFP2 can have a pronounced effect on cellular morphology and actin cytoskeleton organization. While at the population level these effects are largely dependent on transduction conditions (MOI and duration of expression), at the single cell level Lifeact-induced side effects display large heterogeneity, being predominantly dependent on the amount of peptide expressed by each cell.

### Lifeact-induced effects extend to other cytoskeletal networks and the nucleus

**Figure 3:**
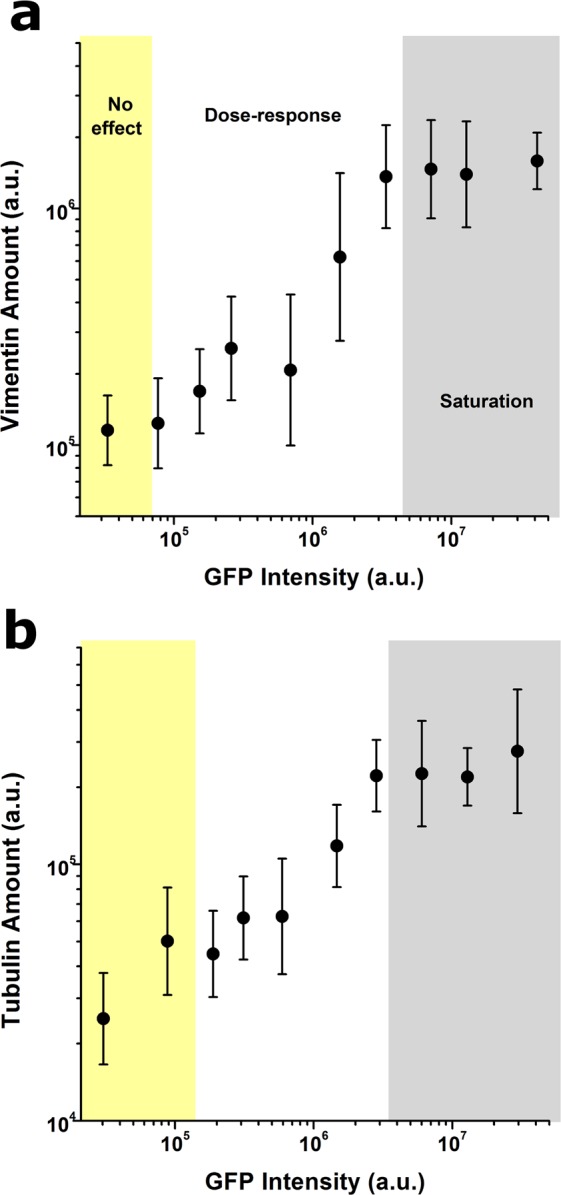
Lifeact-driven effects extend to non-actin-based cytoskeletal networks. Quantification of Lifeact effects on intermediate filaments assembly (**a**) and microtubule assembly (**b**). Values for >40 cells were pooled together to compute each individual data point. Data is presented as geometric mean, error bars indicate geometric standard deviation. Background colours indicate the regimes where cells display no Lifeact-induced effect (yellow background), a dose-response trend (white background) and a saturation plateau (gray background), as identified from analyses of peak changes in variability in the neighbourhood of each point for each parameter plotted.

Having confirmed the marked effects on whole cell morphology and stress fibre architecture induced by Lifeact expression, we chose to focus on Lifeact-TagGFP2 adenoviral transduction on hMSC and we next investigated cellular components with a strong link to the actin cytoskeleton, such as microtubules and intermediate filaments. We limited our protocol to MOI 1000 and 5 days post transduction -to maximise the range of Lifeact expression levels- and replaced TRITC-phalloidin staining with antibodies against tubulin and vimentin. Surprisingly, we found that increased levels of Lifeact expression were associated with a build-up in the microtubule and intermediate filament networks (Fig. 3). Given the close interconnectedness between the three cytoskeletal networks^14^, we hypothesise that alterations in tubulin and vimentin assembly are a secondary result from the effects of Lifeact on cell spread area, rather than a direct interaction between Lifeact peptides and tubulin or vimentin monomers.

**Figure 4:**
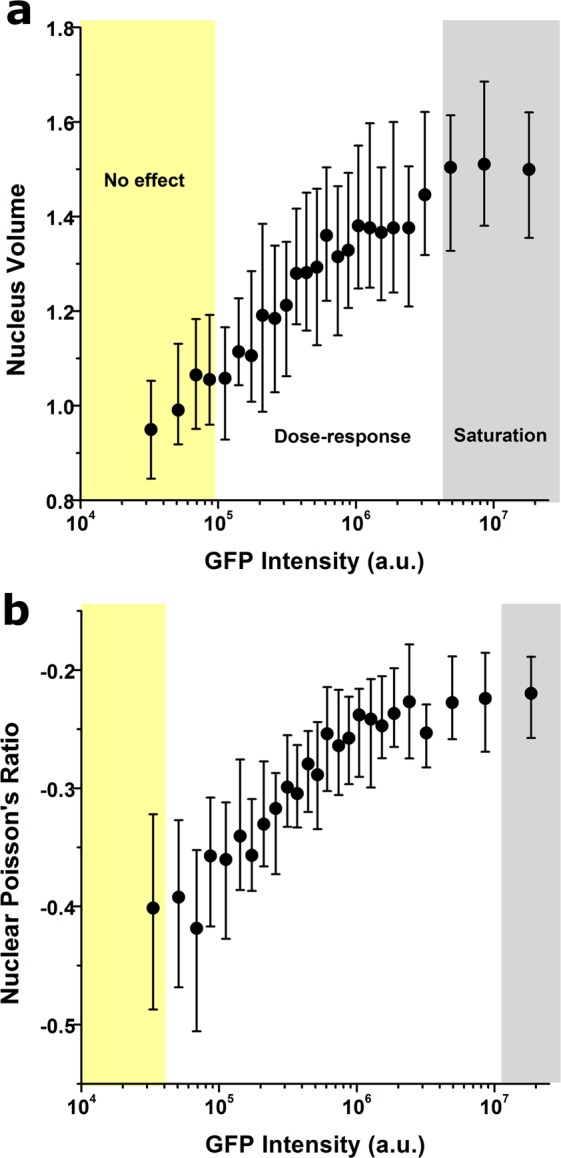
Lifeact-driven effects modulate nuclear state. Quantification of Lifeact effects on nuclear volume (**a**) nuclear Poisson’s Ratio (**b**). Values for >40 cells were pooled together to compute each individual data point. Data is presented as mean, error bars indicate standard deviation. Background colours indicate the regimes where cells display no Lifeact-induced effect (yellow background), a dose-response trend (white background) and a saturation plateau (gray background), as identified from analyses of peak changes in variability in the neighbourhood of each point for each parameter plotted.

We additionally investigated if Lifeact could also influence the nucleus, since nuclear structure is coupled to cytoskeletal organization and cellular morphology. Based on DAPI images from our previous transduction experiments, we quantified changes in three-dimensional nuclear shape, mechanical attributes and chromatin condensation state^14^. As before, we observed that Lifeact expression altered nuclear state, giving raise to nuclei that were up to 1.5 times larger in volume and less auxetic (Fig. 4), while chromatin condensation remained unaffected (not shown). Again, we hypothesize that the effects of Lifeact on the nucleus are a secondary result of alterations in cellular morphology and cytoskeletal architecture^14^. Together, our results uncover for the first time that Lifeact-induced artefacts on the actin cytoskeleton may have knock-on effects that extend into other critical cellular structures.

### Lifeact-induced effects on the cytoskeleton lead to altered cell biophysical behaviour

**Figure 5:**
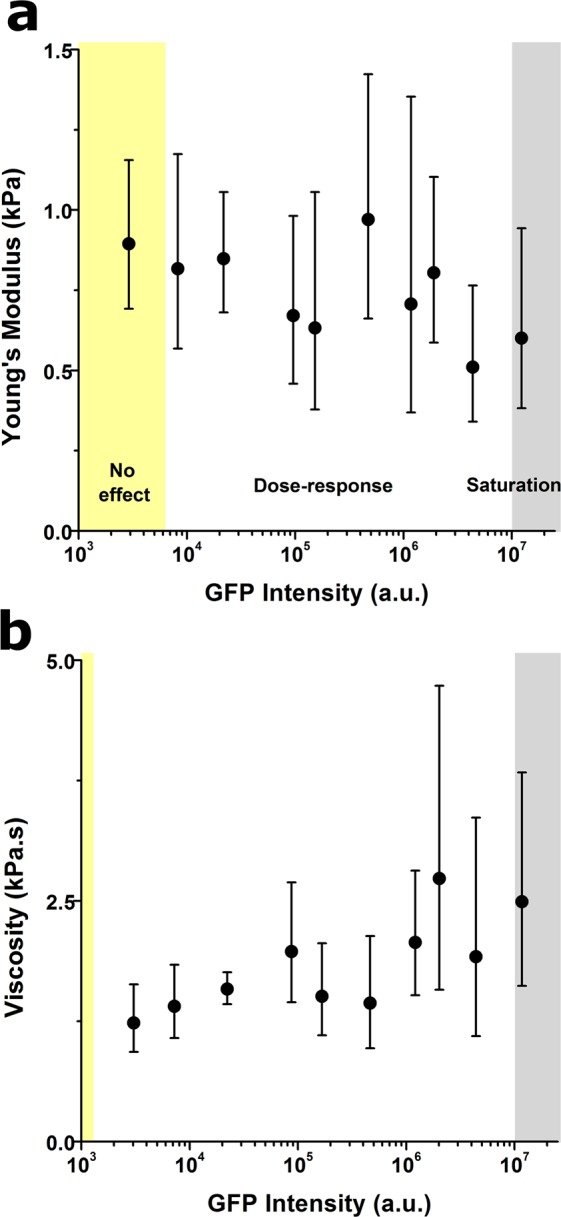
Lifeact expression alters cellular mechanical properties. Lifeact dose dependent effects on cell stiffness (**a**), and viscosity (**b**) Values for >10 cells were pooled to compute each individual data point. Data is presented as geometric mean, error bars indicate geometric standard deviation. Background colours indicate the regimes where cells display no Lifeact-induced effect (yellow background), a dose-response trend (white background) and a saturation plateau (gray background), as identified from analyses of peak changes in variability in the neighbourhood of each point for each parameter plotted.

Having established the multiple effects of Lifeact on cellular structures, we moved to examine their impact on cell biophysical behaviour. First, we used atomic force microscopy to probe the nanomechanical properties of Lifeact-transduced cells. Our results showed a mild decrease in cellular stiffness at very large peptide concentrations together with a steady dose-response increase in cellular viscosity (Fig. 5). These results were initially surprising, as we have previously shown a strong correlation between F-actin assembly and cellular stiffness^13^. Nevertheless, it’s worth stressing that cells with very large levels of Lifeact expression displayed thick fibres disjointed from each other (cell #2 in Suppl. Fig. 7), sometimes leaving between them large cell areas devoid of any actin-rich structure. This scenario is thus very different from the previously described nematic phase of actin organization^15^ (cell #1 in Suppl. Fig. 7) and may rather resemble the liquid-like behaviour of actin structures recently observed *in vitro* after coalescence and shortening of actin bundles^16^.

**Figure 6:**
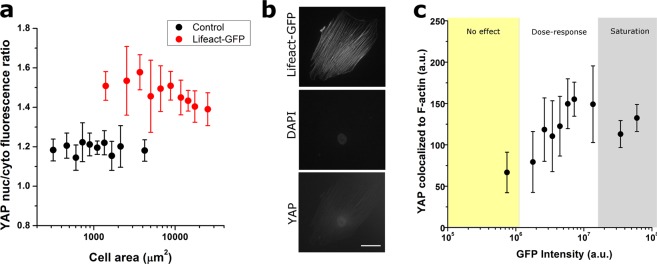
Lifeact expression alters intracellular localization of YAP. Ratio of nuclear to cytoplasmic YAP localization according to Lifeact-TagGFP2 cellular fluorescence (**a**) and cell area (**b**). In (**b**), black symbols correspond to cells not transduced (control) and red symbols correspond to cells transduced with Lifeact-TagGFP2. (**c**) Example cells displaying localization of YAP staining to Lifeact-containing stress fibres. The cell on the left was transduced with Lifeact and the cell on the right was not transduced. After fixation, cells were stained with DAPI (middle panels) and against YAP (bottom panels). Scale bar is 50 µm. (**d**) Average pixel intentisty of YAP fluorescence colocalized to Lifeact-containing stress fibres is dependant on the total amount of Lifeact expressed in the cell. Data is presented as mean, error bars indicate standard deviation. For (**a**) and (**d**), background colours indicate the regimes where cells display no Lifeact-induced effect (yellow background), a dose-response trend (white background) and a saturation plateau (gray background), as identified from analyses of peak changes in variability in the neighbourhood of each point for each parameter plotted. Values for >12 cells were pooled to compute each individual data point.

Increasing evidence points towards the YAP/TAZ pathway as a crucial regulator of cellular mechanosensing in stem cells^17^. In particular, the translocation of YAP into the cell nucleus constitutes a hallmark of increased intra or extracellular forces that are transmitted through the cytoskeleton and to the nucleus^18^. Accordingly, we set to quantify whether YAP intracellular localization would be affected by Lifeact transduction, as a second evidence of altered cell biophysical properties. To this end, we quantified the ratio of nuclear to cytosolic YAP and observed that cells with higher Lifeact-TagGFP2 expression had lower amount of YAP in the nucleus when compared to weakly-transduced cells (Fig. 6a). Furthermore, we explored whether the ratio of nuclear to cytosolic YAP correlated with cell spread area, as found by others^19^. In control cells (not transduced) we found a constant value of nuclear to cytosolic YAP ratio that was not modulated by cell area (Fig. 6b). Conversely, for cells transduced with Lifeact, nuclear to cytosolic YAP ratios were overall larger, and they tended to decrease with increasing cell area (Fig. 6b). This behaviour is reminiscent of that observed in Fig. 5a for cellular stiffness, and may reflect a mild decrease is intracellular tension with increasing Lifeact expression that then results in decreased nuclear translocation of YAP. Of note, immunostaining images of YAP used for this analysis showed a striking unexpected feature, that is, Lifeact-dense stress fibres appeared to be decorated with YAP, a feature that was not observed in control cells (Fig. 6c). In both cases, the preferred nuclear localization of YAP was preserved. Furthermore, we verified that this observation was not due to bleed-through between the GFP and TRITC fluorescence signals, or unspecificity of the TRITC-tagged secondary antibody used throughout this study (Supplementary Fig. 8). Conversely, our analysis shows that YAP colocalization with F-actin fibres increases with increasing Lifeact expression levels (Fig. 6d).

**Figure 7:**
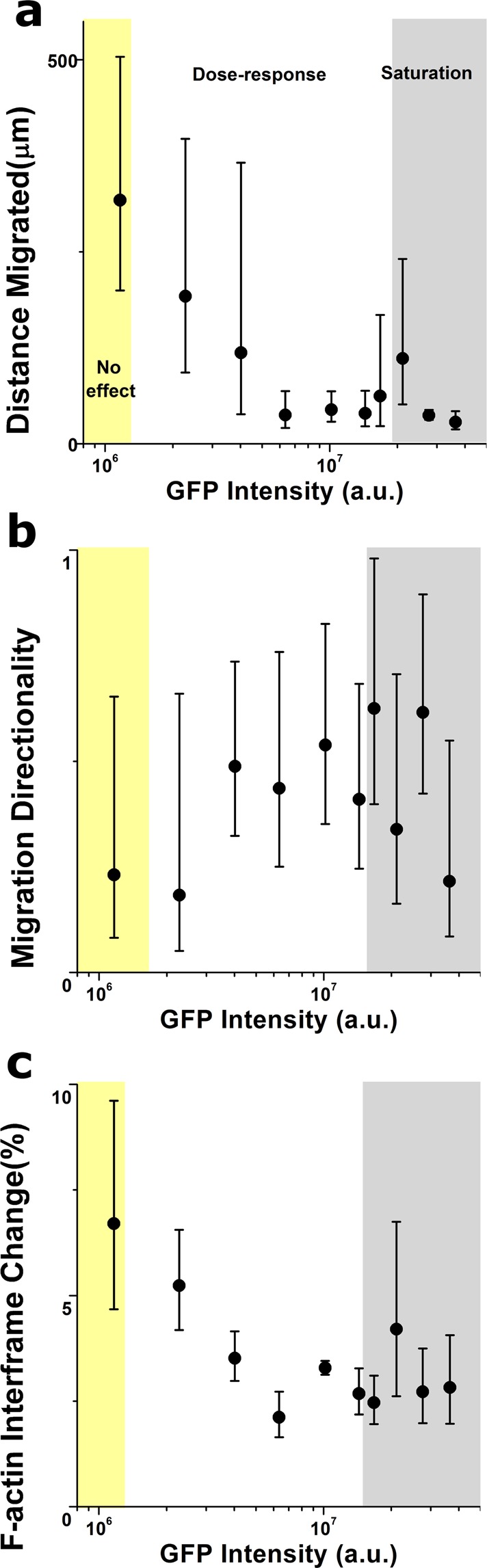
Lifeact expression alters cell migration and F-actin dynamics. Lifeact dose dependent effects on distance migrated (**a**), directionality of migration (**b**) and F-actin inter-frame changes (**c**). Values for >5 cells were pooled to compute each individual data point. Data is presented as geometric mean, error bars indicate geometric standard deviation. Background colours indicate the regimes where cells display no Lifeact-induced effect (yellow background), a dose-response trend (white background) and a saturation plateau (gray background), as identified from analyses of peak changes in variability in the neighbourhood of each point for each parameter plotted.

As a third biophysical behaviour, we evaluated whether Lifeact expression would affect cell motility by performing long-term live cell imaging 5 days post-transduction. Individual cells were tracked by acquiring fluorescence images of the GFP channel every 10 minutes over a period of 18 hours and the resulting videos were later analysed using the same image analysis pipeline as before. In addition to the parameters describing cytoskeletal organization presented above, we also computed the total distance migrated by each cell along with the directionality of migration (Fig. 7). We found that cells displaying low Lifeact expression migrated for longer distances in a less directed fashion. Conversely, cells with intermediate Lifeact expression tended to exhibit shorter but directionally-persistent trails, consistent with our previous finding that these cells tend to display more aligned stress fibers (Supplementary Fig. 4d, Supplementary videos). Finally, cells with very high levels of Lifeact expression exhibited severely impaired migration, remaining quasi-static and erratic in their displacements. Of note, cells that had lower Lifeact expression did reorganize their cytoskeleton to a larger extend in the timeframe of minutes, as shown by the frame-to-frame changes in F-actin assembly (Fig. 7c). Accordingly, we hypothesize that the impaired migration displayed by cells expressing high levels of Lifeact expression is due to reduced F-actin dynamics when reorganizing their cytoskeleton.

### Impaired cofilin binding to F-actin as an underlying mechanism for the Lifeact-induced aberrations in actin organization and dynamics

**Figure 8:**
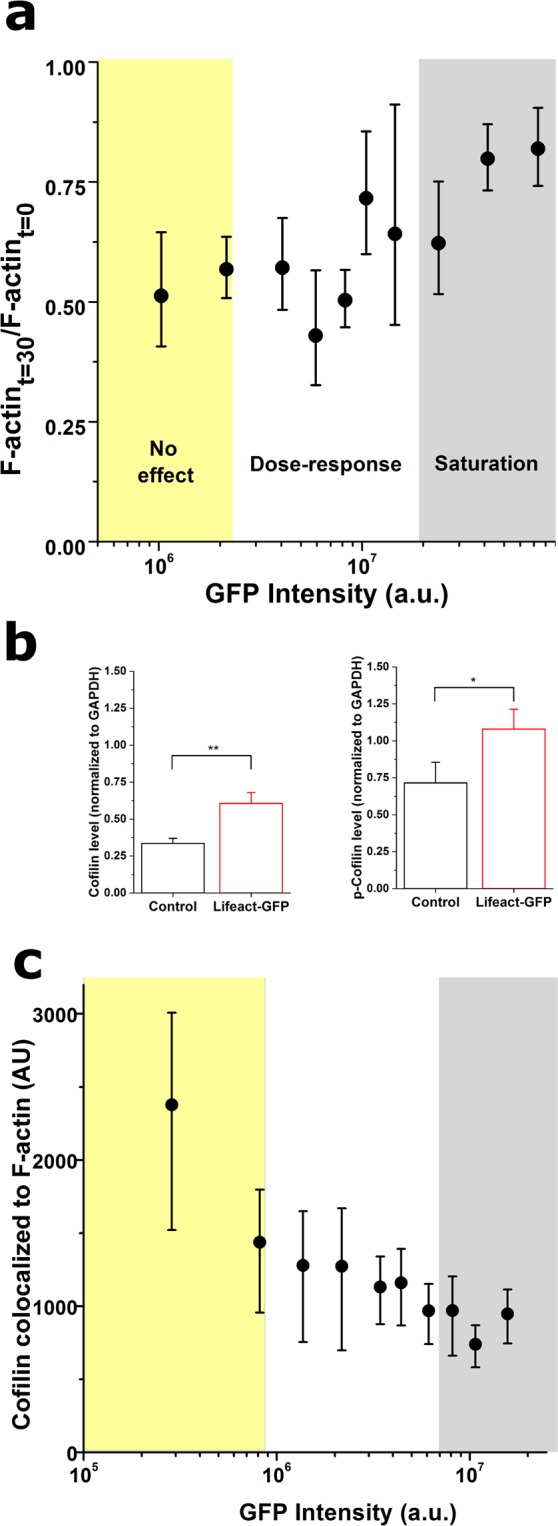
Lifeact expression alters cofilin activity. (**a**) Lifeact dose dependent effects on F-actin disassembly after 30 min of Latrunculin A (0.075 µg/ml) treatment. (**b**) Western blot results for cofilin and p-cofilin expression levels relative to GADPH. (**c**) Lifeact dose dependent effects on fluorescence intensities of cofilin colocalized to F-actin fibres. For (**a**) and (**c**), data is presented as median, error bars indicate Q1-Q3, N > 100 cells; for (**b**) data is presented as mean, error bars indicate standard deviation, N = 3 repeats.

Finally, we set out to pinpoint the potential mechanism by which Lifeact alters F-actin organization and dynamics. Cofilin was identified as a plausible key player, since Lifeact has been suggested by others to impair actin filament severing by cofilin both *in vitro* and in yeast cells^6^. We thus carried out several experiments to assess if and how Lifeact expression led to reduced cofilin activity. On the one hand, we followed the procedure devised by Hotulainen *et al*., which elegantly show that the G-actin sequestering drug Latrunculin A (LatA) fails to depolymerize the actin cytoskeleton when cofilin activity is impaired^20^. We incubated Lifeact-transduced cells with LatA and simultaneously conducted live-cell fluorescence imaging for 30 minutes at 2-minute intervals. By measuring the relative drop in F-actin amount during treatment, we verified that Lifeact reduced LatA-induced cytoskeletal depolymerisation in an expression-dependent manner (Fig. 8a). While this experiment suggested that Lifeact inhibits cofilin activity, it did not identify whether the underlying mechanism is associated with chemical inactivation of cofilin (via phosphorylation at serine residue 3)^21^ or conformational changes of the f-actin filament upon Lifeact binding that prevent cofilin binding^2,6^. Accordingly, we performed western blot measurements of cofilin and p-cofilin expression levels for cell populations transduced with Lifeact or controls (Fig. 8b, Supplementary Fig. 9). Cells transduced with Lifeact displayed 81% increase in overall cofilin expression, while the expression levels of p-cofilin increased only by 51%. Together, these results suggest that Lifeact-transduced cells have higher total amounts of cofilin, and that a lower percentage of said cofilin is in the inactive phosphorylated state. Finally, we performed immunostaining against cofilin to assess whether the drop in cofilin activity was associated with changes in cofilin binding to F-actin. Following the approach devised by Hayakawa *et al*.^22^ using fluorescence image quantification, we measured fluorescence intensity levels of cofilin in pixels previously identified as corresponding to an F-actin fibre, thus obtaining a measure of cofilin colocalization to F-actin. When we produced dose-response curves, we found that cells with higher expression of Lifeact had lower amount of cofilin colocalization (Fig. 8c). Collectively, our results reinforce the hypothesis proposed by Courtemanche *et al*., where Lifeact binding to F-actin induces a conformational change in actin filament structure which is then incompatible with subsequent cofilin binding^6^. This hypothesis should be contextualized with recent findings on the dual activity of cofilin, involving both severing and depolymerisation of actin filaments^23^. Of note, saturation of actin filaments with cofilin dramatically changes their dynamics towards a depolymerisation-prone state from both barbed and pointed ends^23^. Our findings, together with those of others^6,23^, support the hypothesis that prior binding of Lifeact to actin filaments would prevent cofilin saturation of said filaments, thus inhibiting cofilin-induced actin depolymerisation and reducing overall actin filament dynamics.

## Discussion

In summary, our results suggest that Lifeact-TagGFP2 induces dose-response alterations in the actin cytoskeleton, likely stemming from altered cofilin activity and reduced filament dynamics. The effects extend beyond the actin cytoskeleton, also affecting other cytoskeletal structures and impairing the overall biophysical behaviour of cells.

Our findings are more strongly marked in undifferentiated human stem cells, which may be due to a higher capacity to uptake the adenovirally-delivered Lifeact plasmid. Nevertheless, we obtain similar dose-response trends in immortalized cell lines (NIH/3T3 and COS-7), thus expanding the range of cells types were Lifeact has been shown to induce aberrant morphologies^8,9,24^. It is worth stressing that, in our hands, Lifeact affects different cells types to a different degree, and some cytoskeletal features more strongly than others. An illustrative example of a trend being missing is the parameter stellate factor for COS-7 cells (Supplementary Fig. 6b, blue symbols). Stellate factor measures the tendency of a cell to display filopodia projections or protuberances (high value of stellate factor). Indeed, COS-7 cells are rather smooth in their perimetral appearance, and don’t typically extend protrusions. Being that the case, it is difficult to see a trend towards decreasing this value, because even in control conditions this value is low to begin with. Nevertheless, we note that the dose-response trends are typically preserved between cell types, thus suggesting a common origin of the observed changes. The Lifeact plasmid we used included a CMV promoter, which has been shown by others to induce milder aberrations than pBABE and CAG^8^. Of note, our results using a recombinant Lifeact-TagGFP2 protein show that the effect of Lifeact is similar regardless of the way in which the DNA (or protein) is delivered and expressed into the cell. Similarly, the GFP tag used (TagGFP2) is a next-generation monomeric fluorescent protein, again being linked to milder aberrations than other dimerization-prone GFP tags^8^. Our results are thus obtained in conditions identified by others as conductive to fewest aberrant morphologies in terms of choice of promoter and fluorescence protein tag used. In spite of that, we find a dose-response effect at all MOI and conditions used, thus raising concerns on the use of Lifeact as a cytoskeletal marker.

Given that the effects of Lifeact in cytoskeletal organization exhibit a dose-response behaviour with a saturation plateau, our results bring new light to the difficult compromise during transduction optimization, that is, maximising the number of transduced cells while reducing the number of cells which are either dead or with aberrant morphologies. Contrary to expected, for all transduction protocols that we tested, the number of cells that are transduced but not aberrant is constant and much lower than anticipated (<20%) (Supplementary Fig. 10). Lifeact transduction protocols found in published literature vary to a certain degree between laboratories and also according to cell lines used. It may then be difficult to judge, when optimizing a transduction protocol, whether experiments are being carried out in non-artifactual conditions based only on MOI estimates. We propose an alternative approach, that is, that the presence of few clearly obvious aberrant cells (gray bars in Supplementary Fig. 10) should be used as a tell-tale sign that a large percentage of cells are within the dose-response regime (white bars in Supplementary Fig. 10) and that few cells will be truly non-artifactual. We note here that transduced cells that display minor aberrations are likely to go unnoticed to the naked eye during the course of an experiment. Selection of these cells in a study will lead to experimental bias or lack of reproducibility with results obtained using other live cell actin probes. Prior to performing experiments, it is important researchers establish a reliable protocol to identify and select only suitable cells within the whole population of heterogeneously transduced cells. Similarly, it would be advisable to report the percentages of not-affected, aberrant and grossly-aberrant cells within the cell population for any given transduction protocol used in a study. Preliminary tests based on co-staining with an actin marker such as phalloidin and image quantification at the single cell level can provide this type of information in a swift manner. With this study, we hope to start an active discussion on what are the limits of suitability of our current live-cell cytoskeletal reporters. This is a timely and much-needed debate, especially with the advent of other actin reporters, such as SiR-actin, Utrophin or F-tractin, which may display similar associated issues.

## Methods

### Cell culture, Lifeact-TagGFP2 transduction and immunostaining

Unless stated otherwise, all chemicals and reagents were obtained from Sigma. The majority of measurements were performed in human bone marrow derived mesenchymal stem cells (Promocell), while additional measurements were performed in NIH/3T3 and COS-7 cells. Cells were maintained in culture medium consisting of low glucose Dulbecco’s Modified Eagle Media (Gibco) supplemented with 10% foetal bovine serum, and 100 U/ml Penicillin- 100 µg/ml Streptomycin. hMSCS were additionally supplemented with 10 ng/ml fibroblast growth factor (Peprotech). Cells were kept in tissue culture flasks and cultured at 37 °C and 5% CO_2_. Mesenchymal stem cells were used between passages 5 and 9. Lifeact-TagGFP2 transductions were performed using commercial rAV-CMV-LifeAct-TagGFP2 Adenoviral Vectors (Ibidi) according to supplier’s instructions, by addition of viral transduction reagent volumes required to achieve the desired MOI (i.e. 100, 300, 600 or 1000) on each sample. After the initial 18 hours of incubation for vector uptake, media containing viral particles was exchanged. Cell samples were allowed to express Lifeact-TagGFP2 for a total of 1, 3, 5 or 7 days prior to fixation. The pCMV-EGFP plasmid was a kind gift from Dr Julien Gautrot. For experiments using recombinant Lifeact-TagGFP2 protein, Lifeact-TagGFP2 peptides and proprietary Fuse-it-P intracellular protein delivery kits were acquired from Ibidi and prepared according to instructions. Briefly, hMSCs were seeded into coverslips inside 6-well TCP vessels, three days before experiments. Lyophilised peptides were reconstituted in sterile water, and further diluted in 20 mM HEPES buffer to a concentration of 0.1 mg/ml. Fuse-it-P was loaded with peptides by following supplier’s instructions. Cells were washed in PBS, and 1 ml of 1:50 fusogenic mixture was dispensed to each well. After incubation for 5 minutes at 37 °C, fusogenic mixture was replaced with cell culture medium and returned to an incubator. Cell samples were fixed after 6 hours, to mitigate toxicity effects, stained and imaged as before. All live cell experiments (migration, AFM and Latrunculin-A treatment) were conducted on cells transduced at MOI 1000, at 5 days post transduction. The same conditions were used for NIH3/3 and COS-7 cells. At least 3 independent transductions were performed for each set of experiments.

For live cell imaging experiments, cells were directly plated onto 6-well plates and cultured in FBS and antibiotic supplemented Flurobrite-DMEM imaging specific media (Thermofisher). For AFM measurements, cells were plated in petri dishes and imaging media were supplemented with 50 mM HEPES. For immunostaining experiments, cells were sparsely seeded onto serum coated coverslips inside sterile petri dishes at least 1 day prior to transductions. In brief, cells were fixed by treatment with 3.7% paraformaldehyde in PBS for 15 min and permeabilised for 5 min in 0.25% Triton X-100. To visualize simultaneously F-actin via Lifeact and Phalloidin, cells were stained with phalloidin-TRITC at 2 μg/ml in PBS for 2 hours. For additional immunostaining experiments to visualize other cytoskeletons and proteins, permeabilized cells were treated overnight with primary antibodies against vimentin (1:400 dilution; RV202), α-tubulin (1:50 dilution; TU-02), YAP (1:200, 63.7) and cofilin (1:200; E-8) diluted in goat serum blocking buffer at 4 °C (all antibodies mouse monoclonal from Santa Cruz Biotechnologies). The next morning, the samples were washed with PBS and treated with a TRITC-tagged secondary antibody (1:400 dilution, goat anti-mouse IgG-TRITC, sc-3796) for 1 hour at room temperature. All coverslips were mounted onto glass slides using ProLong® Gold Antifade Mountant containing DAPI (Thermo Fisher). Control samples were cultured and stained in parallel to transduced cell cultures, but without having been subjected to the transduction protocol.

### Quantification of cell morphology, cytoskeletal structures and nuclear state from fluorescence images

All fixed samples were imaged using an inverted epifluorescence microscope (Leica DMI4000B) with a x20/0.50 NA objective lens and a CCD camera (Leica DFC300FX). Cells were sequentially imaged on the DAPI (nuclei), TRITC (phalloidin/antibody staining), and FITC (Lifeact-TagGFP2) channels. The algorithm for single-cell quantification of cytoskeleton structures has been described in previous publications^13,14^. The coded algorithm (CSKMorphometrics) has been implemented in MATLAB (Mathworks) and can be found at the File Exchange repository at MATLAB central site. In brief, the quantification of cell morphology and cytoskeleton configuration is based on three steps: (1) initial fibre segmentation, (2) fibre refinement, and (3) determination and subtraction of background. These steps output a variety of maps representing either the brightness of segmented fibres or local fibre orientation, that allow subsequent estimation of morphometric parameters for individual cells. This information is assembled into 14 descriptors (Supplementary Information), e.g. cell spread area, total fibre amount. For the present study, we use the term ‘fibre amount’ to signify the amount of protein organized in fibres, that is, identified by the pipeline as part of the segmented cytoskeleton in the raw image.

Quantification of nuclear features to estimate relative nuclear mechanical parameters is described elsewhere^14^. With this method we process DAPI-stained nuclei images to quantify nuclear volume, Poisson ratio and chromatin state in individual cells.

Finally, the total intensity from GFP images belonging to individual cells was used as a metric for intracellular Lifeact amount to produce graphs correlating cellular morphometrics with peptide expression. Total GFP intensity was measured by adding up the fluorescence intensity measured for all pixels within the outline of a cell, once background intensity was subtracted. To statistically identify the three regimes in the dose-response curves, namely a no effect regime, a dose-response regime, and a saturation plateau, threshold points were calculated across all parameters by adapting a method previously developed by us and based on the ratio of variances (RoV)^25,26^ around each point of a DRC (Figs 2–8 and Supplementary Fig. 4). Briefly, a test parameter RoV is defined as $RoV{\rm{i}}=\frac{var(di+1:{\rm{d}}i+N)}{var(d{\rm{i}}-N:d{\rm{i}}-1)}$, i.e. the ratio of the variances computed in two N-sized small windows to each side of every point *i* in each DRC. Peaks in RoV displaying regions of high variability in the data, signifying a transition between regimes, were identified in each DRC curve. Two global GFP intensity values corresponding to the transitions point to dose-response and saturation regimes were obtained by averaging out all threshold GFP intensities obtained in Figs 2–8 and Supplementary Fig. 4. The values for the two global GFP intensity thresholds are included in Supplementary Fig. 2 and were used to sort individual cells into the 3 regimes depicted in Supplementary Fig. 10.

### Quantification of Nuclear/Cytosolic ratio of YAP

Nuclear/Cytosolic ratio of YAP was assessed as previously described by others^18^. Briefly, we measured the average fluorescence intensities of YAP staining in the nucleus and in an annular region with equal size in the cytosol immediately adjacent to the nuclear region, and computed their ratio.

### Western Blotting

Cells were washed with chilled PBS and lysed in RIPA buffer for 15 min on ice. The total protein concentration was determined by the BCA assay. Cell lysates were mixed with Laemmeli buffer and denatured by heating at 100 °C for 5 min. Proteins were separated by SDS–PAGE and transferred onto a nitrocellulose membrane. Membranes were blocked in 5% dry milk for 1 h, followed by incubation with primary antibodies for cofilin (1:125, E-8, Santa Cruz), p-cofilin (1:250, E-5 Santa Cruz) and control glyceraldehyde 3-phosphate dehydrogenase (GAPDH) (1:500, 0411, Santa Cruz) over night at 4 °C. Excess of antibody was removed by washing with PBST three times and the secondary antibody donkey anti-mouse (IRDye® 680RD Donkey anti-Mouse IgG (H + L), [P/N 926-68072]; 1:10000) was added for 1 h at room temperature in dark. The proteins recognized by the antibody were visualized by chemiluminescence. ImageJ was used to quantify the intensity of cofilin, p-cofilin and GAPDH protein bands from each blot.

### Migration and cytoskeleton disassembly experiments

For migration and Latrunculin-A treatment experiments, live-cell imaging was performed under temperature and CO_2_ controlled environment, using an incubator-encased epifluorescence imaging system (Lumascope 720, Etaluma) at 20x magnification. Transduced cells were cultured inside 6-well plates until the time of imaging. Individual cells were continuously tracked for 18 hours at 10-minute intervals, and imaged in the FITC channel. To produce Supplementary videos of long term behaviour in Lifeact-TagGFP2 expressing cells, imaging was conducted under similar conditions using 10x magnification for a period of 4 days, sampled at 1-hour intervals. Control cells remained untransduced for the duration of the experiment. Other conditions consisted of cells transduced at MOIs of 250 or 500.

To characterize migration patterns, every frame on the 18 hours time-lapse video pertaining to the Lifeact-TagGFP2 channel was analysed using the formerly described image processing algorithms. The positions of cell centroids were tracked from masks of instantaneous cell shape and used to quantify total distance migrated. Migration directionality was defined as the ratio between net cell displacement (the euclidian distance between starting and ending centroid positions) and the overall distance travelled by the cell, as $MD=\frac{d({P}_{t=0},{P}_{t=T})}{{\sum }_{i=0}^{T}\,d({P}_{t=i},{P}_{t=i+1})}$. F-actin interframe change was calculated comparing values of F-actin (FA) between successive frames, as $IF{C}_{i}=100\cdot \frac{F{A}_{i+1}-F{A}_{i}}{F{A}_{i}}$. For cytoskeleton disassembly studies, cells were imaged for 30 minutes at 2-minute intervals immediately upon addition of Latrunculin A (0.075 µg/ml) to the culture medium. F-actin disassembly was quantified as $100\cdot \frac{F{A}_{t=0min}-F{A}_{t=30min}}{F{A}_{t=0min}}$

### Determination of cellular stiffness and viscosity with atomic force microscopy

All measurements of cell mechanics were performed on a Nanowizard 4 (JPK), integrated with an Axio Observer Z.1 epifluorescence microscope with Plan-Apochromat lenses (20×) equipped with a cooled CMOS camera (Orca Flash 4). Cells were probed using gold-coated rectangular cantilevers (0.03 N/m nominal spring constant) with pyramidal tips (12 µm high with 35° half cone angle, supplied by BudgetSensors). Experiments were conducted on petri dishes mounted on a heating accessory to maintain cells at 37 °C. AFM experiments were conducted for a maximum of 1 hr per petri dish. Prior to measurement, the cantilevers were allowed to thermally equilibrate fully submerged in cell media. The cantilever sensitivity was calibrated in contact mode on a bare region of the container, following which the cantilever was moved a minimum of 500 µm from the surface to calibrate the force constant using thermal fluctuations. We identified individual adherent cells exhibiting varied levels of GFP expression and recorded a fluorescence image of the GFP channel at 20× magnification before measuring cell mechanics. Imaging parameters (exposure time and gain) were kept constant for all experiments. AFM measurements were performed using JPK’s QI mode, which rapidly acquires force-curves generating a detailed image of the topography and mechanical properties of the sample. For each measurement we selected a region of 100 by 100 µm (32 × 32 force curves) ranging from lamellar and cytosolic to nuclear regions of the cell. Force curves had a z-length of ~10 µm, extension speed of 125 µm/s and a setpoint of 3–5 nN.

Data analysis of the force-displacement curves was carried out using the BECC model for thin adherent cells on a stiff substrate^25^ using a pipeline written in MATLAB as previously described^26^. Cellular viscosity was computed using the same force-displacement curves following the method outlined by Rebelo *et al*.^27^.

### Statistical analysis

Statistical tests were produced with the OriginLab analysis software. Population results were plotted as box charts presenting median values and first and third quartiles, with error bars indicating the 1^st^ and 99^th^ quartiles. Single cell results were expressed either as means or geometric means with error bars representing interquartile range. Two-way ANOVA tests were used to establish the significance of concentration and time effects on the levels of Lifeact expression and of morphological alterations of cell populations. Dunnett’s post-hoc tests where used to determine significant differences between the control group (no transduction) and groups treated with increasing MOIs for each day measured.

## Supplementary information

Supplementary video 1

Supplementary video 2

Supplementary video 3

Supplementary information
