# Author Correction: Lifeact-TagGFP2 alters F-actin organization, cellular morphology and biophysical behaviour

**DOI:** 10.1038/s41598-019-45276-y

**Published:** 2019-06-26

**Authors:** Luis R. Flores, Michael C. Keeling, Xiaoli Zhang, Kristina Sliogeryte, Núria Gavara

**Affiliations:** School of Engineering and Materials Science, Queen Mary University of London, Mile End Road, E1 4NS London, UK

Correction to: *Scientific Reports* 10.1038/s41598-019-40092-w, published online 01 March 2019

The original version of this Article and the accompanying Supplementary Information file contained a repeated error where,

“Lifeact-GFP”

now reads:

“Lifeact-TagGFP2”

