# Supplementary material for: Lifeact-TagGFP2 alters F-actin organization, cellular morphology and biophysical behaviour
Source: Sci Rep. 2019 Mar 1;9:3241. doi: 10.1038/s41598-019-40092-w (PMC6397297; doi:10.1038/s41598-019-40092-w)
Supplement: Supplementary file 4 — Supplementary information [file 41598_2019_40092_MOESM4_ESM.docx]

**Lifeact-TagGFP2 alters F-actin organization, cellular morphology and biophysical behaviour**

Luis R. Flores^1^, Michael C. Keeling^1^, Xiaoli Zhang^1^ , Kristina Sliogeryte^1^ & Núria Gavara^1*^

^1^School of Engineering and Materials Science, Queen Mary University of London, Mile End Road, E1 4NS, London, UK.

^*^e-mail: [n.gavara@qmul.ac.uk](mailto:n.gavara@qmul.ac.uk)

**Supplementary Information**

**Computation of morphometric parameters**

The parameters presented below are measured from fluorescence images of single cells using a custom-build pipeline written in MATLAB (Mathworks). More extensive information can be found in previous publications^13,14^. The pipeline is available upon reasonable collaborative requests.

**Morphology metrics**

**Cell spread area** – sum of pixels contained within the mask of cell shape, converted into metric system (μm^2^).

**Aspect ratio** – Ratio between major and minor axis of cell shape, excluding filopodia

**Stellate factor** – Measurement of the convexity of cell area, larger values indicate the presence of filopodia or a more stellate shape, whereas lower values indicate a more solid shape.

**Cytoskeleton fibre metrics**

**Cytoskeleton amount** – Sum of pixel intensities for all pixels identified as belonging to a fibre

**Thickness** – Average of pixel intensities for all pixels identified as belonging to a fibre. Given that in our imaging conditions that pixel size is larger than the diffraction limit and the thickness of an actin filament, pixel intensity constitutes a good surrogate measure to estimate the number of individual fluorophores bound to a filament and the number of filaments making up a stress fibre.

**Thickness variation** – Variability in the distribution of all pixel intensities for all pixels identified as belonging to a fibre.

**Alignment of fibres –** Using the previously computed orientations for all pixels identified as belonging to a fibre, alignment is defined as 1- circular variance, computed using directional statistics of the distribution of angles. Values close to 1 indicate that the majority of fibres are oriented in the same direction, whereas values close to 0 indicate random orientation of fibres.

**Curvature of fibres** – Similar to alignment, but computed as circular variance for all pixels *within a single fibre*. Values close to 1 indicate fibres that are very curvy, whereas values close to 0 indicate straight fibres.

**Location of fibres** – Radial position where fibres are preferentially found in the cell. A value of 1 indicates the cell edge (closer to the cell periphery), whereas a value of 0 indicates the cell’s centroid (closer to the centre of the cell)

**Fibre spread** – Variance associated with location of fibres in the radial position (see above). A larger value indicates that fibres are well spread through the cell diameter, whereas a smaller value indicates that fibres are preferentially localized in a single radial position.

**Length** – Average length of the fibres in a cell (in µm).

**Length variability** – Variance associated with the length of the fibres in a cell.

**Chirality** – Once the centroid of the cell is identified, the fibre orientation map is converted to compute the relative orientation of each fibre with respect to the cell’s centroid. A value close to 0° indicates that fibres are preferentially pointing in the radial direction (towards the centre of the cell) whereas a value close to 90° indicates that fibres are preferentially pointing in the circumferential direction (in parallel to the cell edge)

**Chirality variability** – Variance associated with chirality measurements


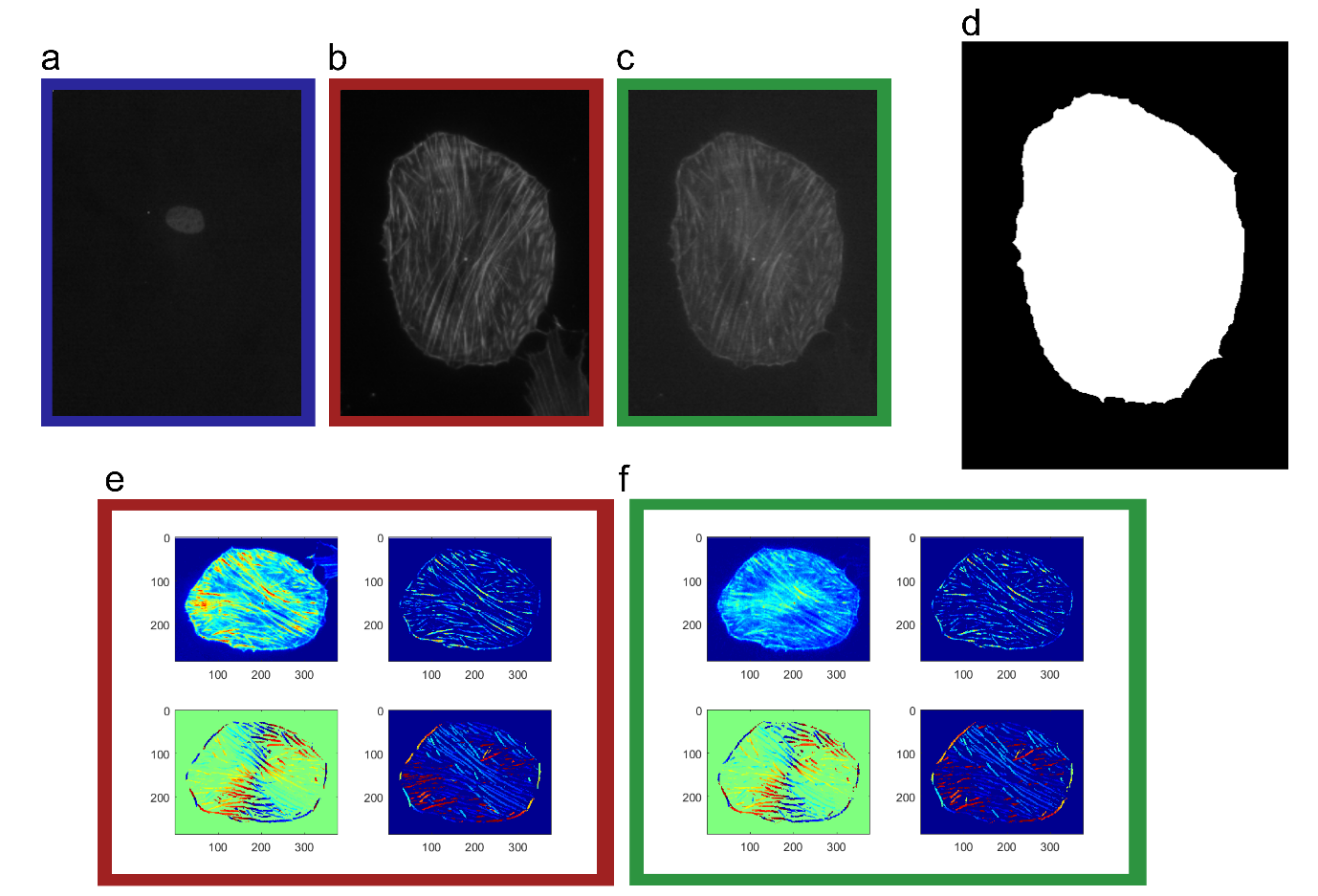


**Supplementary Figure 1** Example inputs and outputs of our image analysis pipeline. DAPI (a), phalloidin-TRITC (b) and Lifeact-TagGFP2 (c) images are provided as inputs to calculate nuclear properties, cytoskeleton morphometric parameters and total GFP intensity, respectively. An individual mask is generated from these images to identify the perimeter of a single cell (d). Comparative examples of cytoskeleton fibre sampling maps from the TRITC channel (e) and GFP channel (f); in cells with intermediate to high Lifeact expression, as displayed, fibre sampling is reasonably similar between the two. Top quadrants represent maps of total intensity and identified fibres, bottom quadrants map angular orientation of fibres.

**Supplementary Table 1** Example cells highlighting the morphological appearance of each computed parameter. For each parameter, two cells corresponding to low and high value ranges are pictured, with specific values included. Lifeact-TagGFP2 fluorescence intensity values are also included for each cell. All cells are stained with phalloidin-TRITC. Scale bars are 30 µm for all cells plotted.

| **Cell spread area (µm^2^)**  0-∞ | 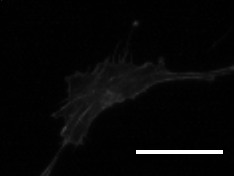  686 µm^2^  Fluorescence Intensity = 2.1·10^4^ | 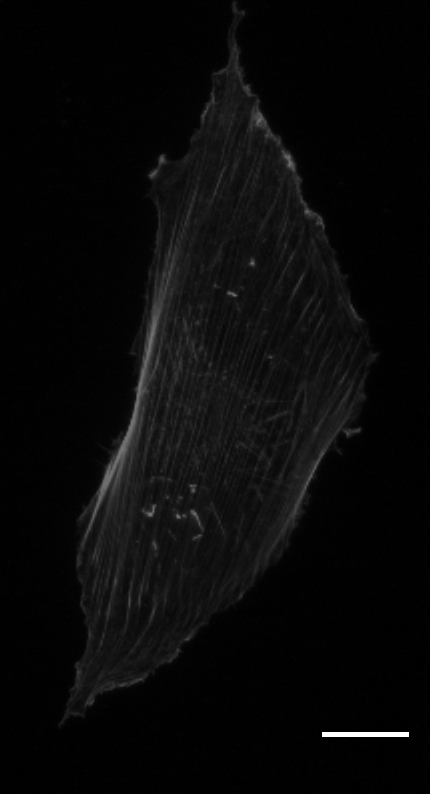  12292 µm^2^  Fluorescence Intensity = 9.1·10^6^ |
| --- | --- | --- |
| **Aspect ratio**  1-∞ | 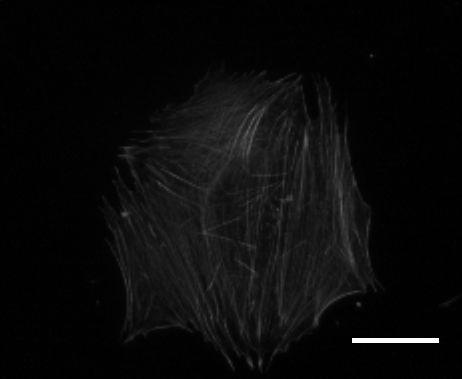  1.13  Fluorescence Intensity = 2.0·10^6^ | 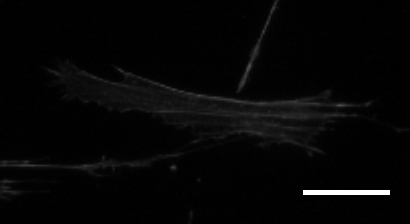  5.89  Fluorescence Intensity = 2.2·10^5^ |
| **Stellate factor**  0-∞  (typically 0.1-0.8) |   0.11  Fluorescence Intensity = 3.0·10^6^ |   0.62  Fluorescence Intensity = 3.4·10^5^ |
| **F-actin amount (AU)**  1-∞ | 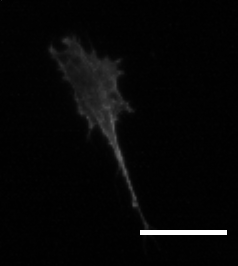  3.1·10^5^  Fluorescence Intensity = 3.4·10^4^ |   1.3·10^7^  Fluorescence Intensity = 1.4·10^6^ |
| **Fibre thickness**  **(AU)**  1-∞ |   65  Fluorescence Intensity = 7.4·10^4^ |   425  Fluorescence Intensity = 7.6·10^6^ |
| **Fibre thickness**  **CoV (%)**  0-∞  (typically 50-200%) |   81%  Fluorescence Intensity = 3.2·10^5^ |   149%  Fluorescence Intensity = 9.1·10^5^ |
| **Fibre alignment**  0-1  (typically 0.6-1) |   0.74  Fluorescence Intensity = 4.4·10^5^ |   0.96  Fluorescence Intensity = 1.4·10^6^ |
| **Fibre curvature**  0-1  (typically 0-0.6) |   0.074  Fluorescence Intensity = 2.3·10^5^ |   0.418  Fluorescence Intensity = 1.3·10^5^ |
| **Location of fibres**  0-1 |   0.336  Fluorescence Intensity = 1.4·10^7^ |   0.898  Fluorescence Intensity = 3.1·10^5^ |
| **Fibre spread**  0-1  (typically 0.1-0.3) |   0.10267  Fluorescence Intensity = 1.8·10^5^ |   0.27147  Fluorescence Intensity = 9.1·10^4^ |
| **Fibre length (µm)**  0-∞ | 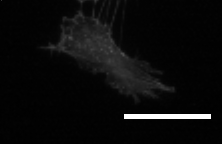  7.4 µm  Fluorescence Intensity = 6.0·10^4^ |   39.4 µm  Fluorescence Intensity = 1.4·10^7^ |
| **Fibre length CoV (%)**  0-∞ |   71.3 %  Fluorescence Intensity = 7.6·10^4^ |   247.7 %  Fluorescence Intensity =1.2·10^7^ |
| **Chirality (deg)**  0-90 |   35.2 deg  Fluorescence Intensity =2.8·10^6^ |   89.6 deg  Fluorescence Intensity =1.4·10^5^ |
| **Chirality variance (deg^2^)**  0-8100 |   239.8 deg^2^  Fluorescence Intensity =2.2·10^5^ |   772.5 deg^2^  Fluorescence Intensity =2.7·10^6^ |





**Supplementary Figure 2** GFP intensity increases in cell populations transfected at higher MOIs and with longer expression time. Box plots represent median values, and first and third quartiles, while error bars represent the 1^st^ and 99^th^ percentiles. N > 2500 cells. Two-way ANOVA test showed significant differences for MOI (p<0.001) and expression time (P<0.001). Dashed lines indicate the thresholds separating the ‘no effect’, dose-response and saturation regimes.


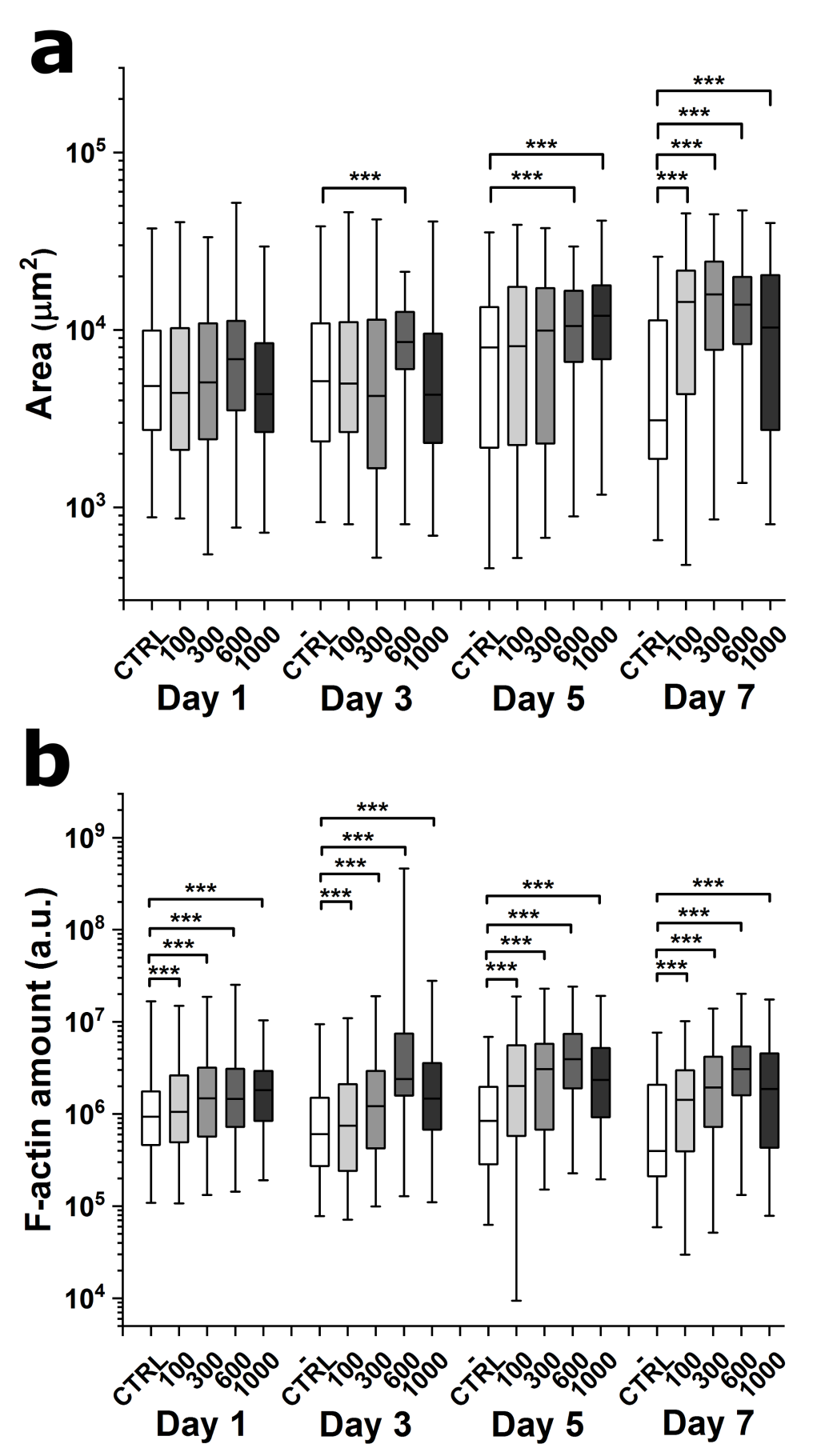


**Supplementary Figure 3** Cell area and stress fibres amount increase in cell populations transfected at higher MOIs and with longer expression time. Box plots represent median values, and first and third quartiles, while error bars represent the 1^st^ and 99^th^ percentiles. N > 2500 cells. Two-way ANOVA tests showed significant differences for MOI (p<0.001) and expression time (P<0.001) for both cell area and stress fibre amount. *** indicates p<0.001 as obtained from Dunnett’s post hoc test against control for each day.

**
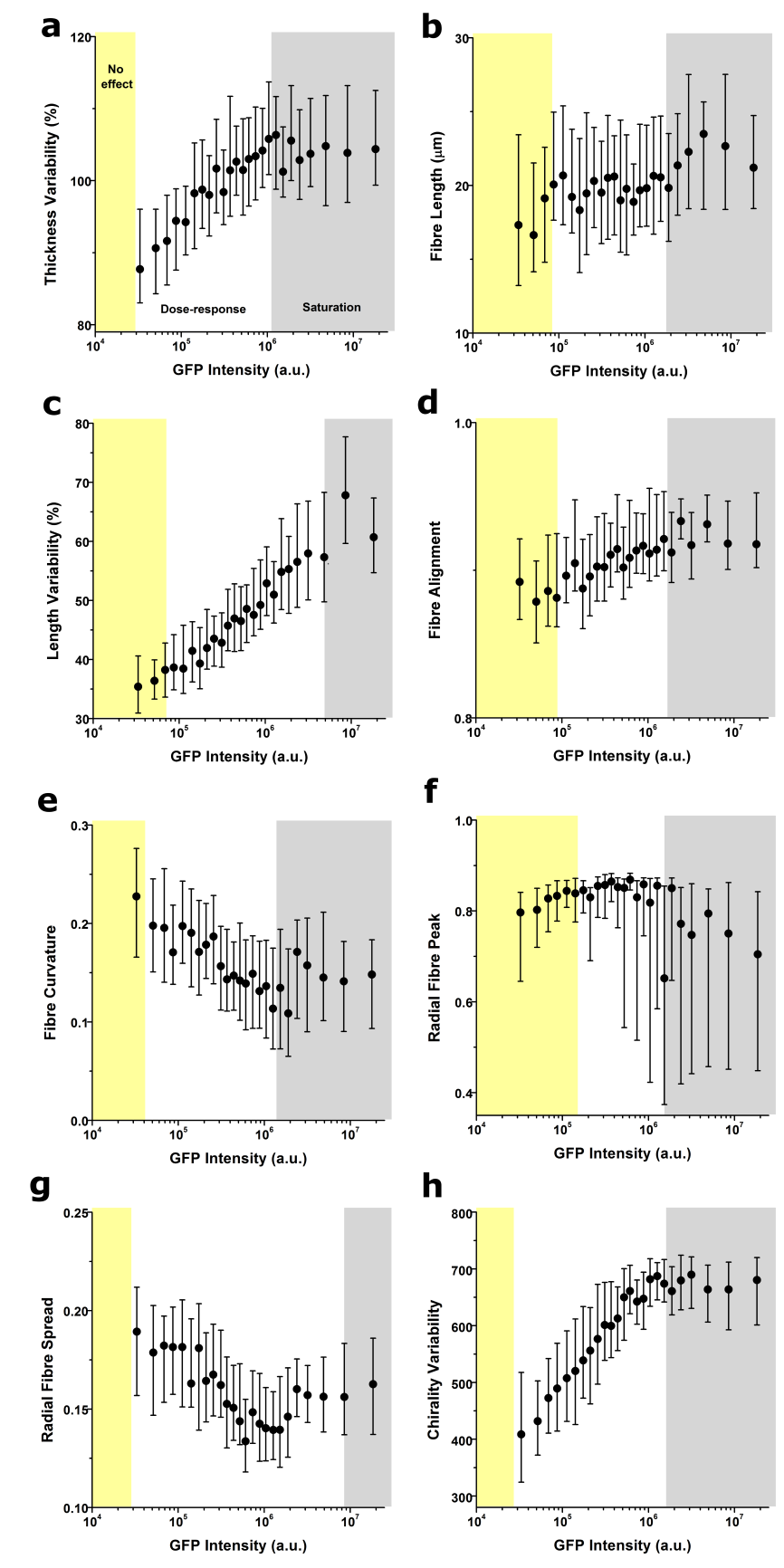
**

**Supplementary Figure 4** Lifeact-TagGFP2 expression affects cellular morphology and cytoskeletal organization in a dose-response manner**.** Dose-response curves quantifying the effect of Lifeact expression in whole cell aspect ratio (a), fibre thickness variability (b), fibre alignment (c) and curvature (d), peak fibre location (e) and spread (f), fibre length (g) and associated variability (h), and variability of chirality of fibres (i). Values for >100 cells were pooled together to compute each individual data point. Data is presented as mean, error bars indicate interquartile range (Q1–Q3). Background colours indicate the regimes where cells display no Lifeact-induced effect (yellow background), a dose-response trend (white background) and a saturation plateau (gray background), as identified from analyses of peak changes in variability in the neighbourhood of each point for each parameter plotted.

**
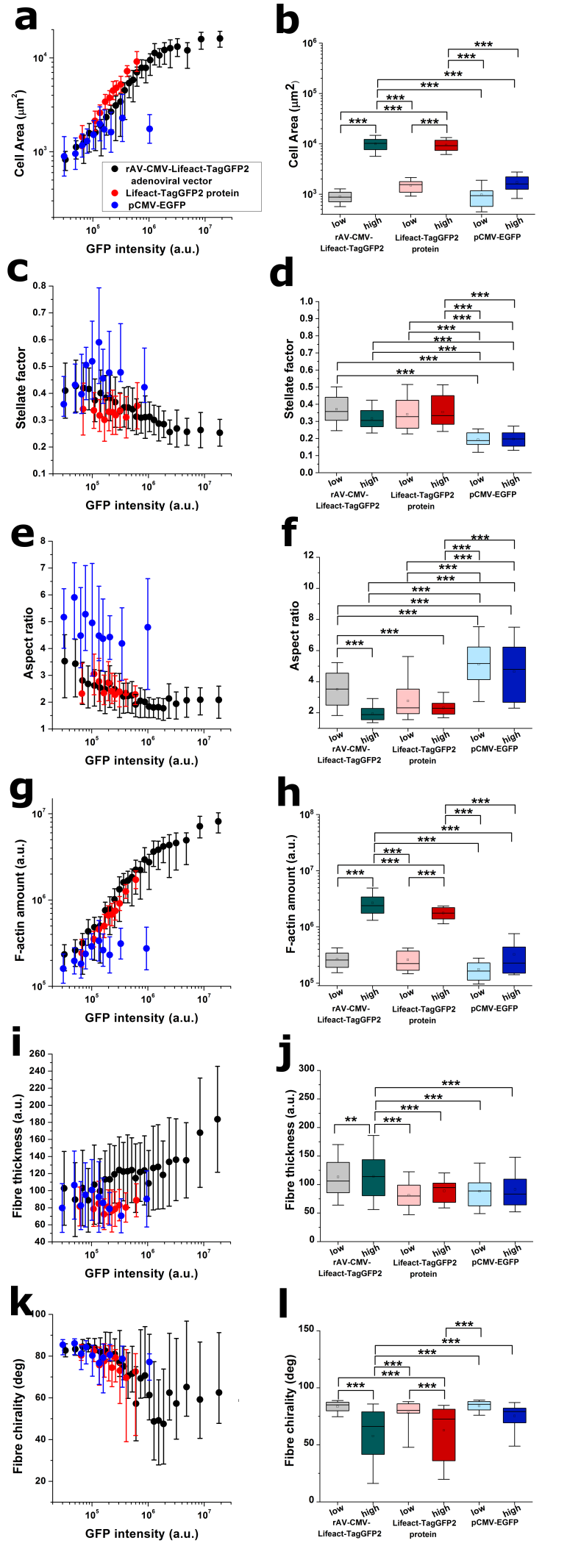
**

**Supplementary Figure 5.** Similar Lifeact-induced effects are observed irrespective of Lifeact intracellular delivery. Dose-response curves quantifying the effect of Lifeact expression when cells are transduced with a rAV-CMV-Lifeact-TagGFP2 plasmid (black), recombinant Lifeact-TagGFP2 protein (red) and pCMV-EGFP (blue). Plotted is cell spread area (a), cell perimeter stellate factor (c), aspect ratio (e), f-actin amount (g), fibre thickness (i) and chirality of fibres (k). Values for >10 cells were pooled together to compute each individual data point. Data is presented as geometric mean (a and g), mean (b and e) or median (c and f) error bars indicate geometric standard deviation, standard deviation or Q1-Q3, accordingly. Right panels (b, d, f, h, j, l) correspond to multiple comparisons between methods of intracellular deliver (black for rAV-CMV-Lifeact-TagGFP2, red for Lifeact-TagGFP2 protein, blue for pCMV-EGFP) for selected low range (3.5·10^4^) and a high range (1·10^6^) of GFP expression. 1-way ANOVA followed by Bonferroni post-hoc test was used, * is p<0.05, ** is p<0.01, *** is p<0.001.

**
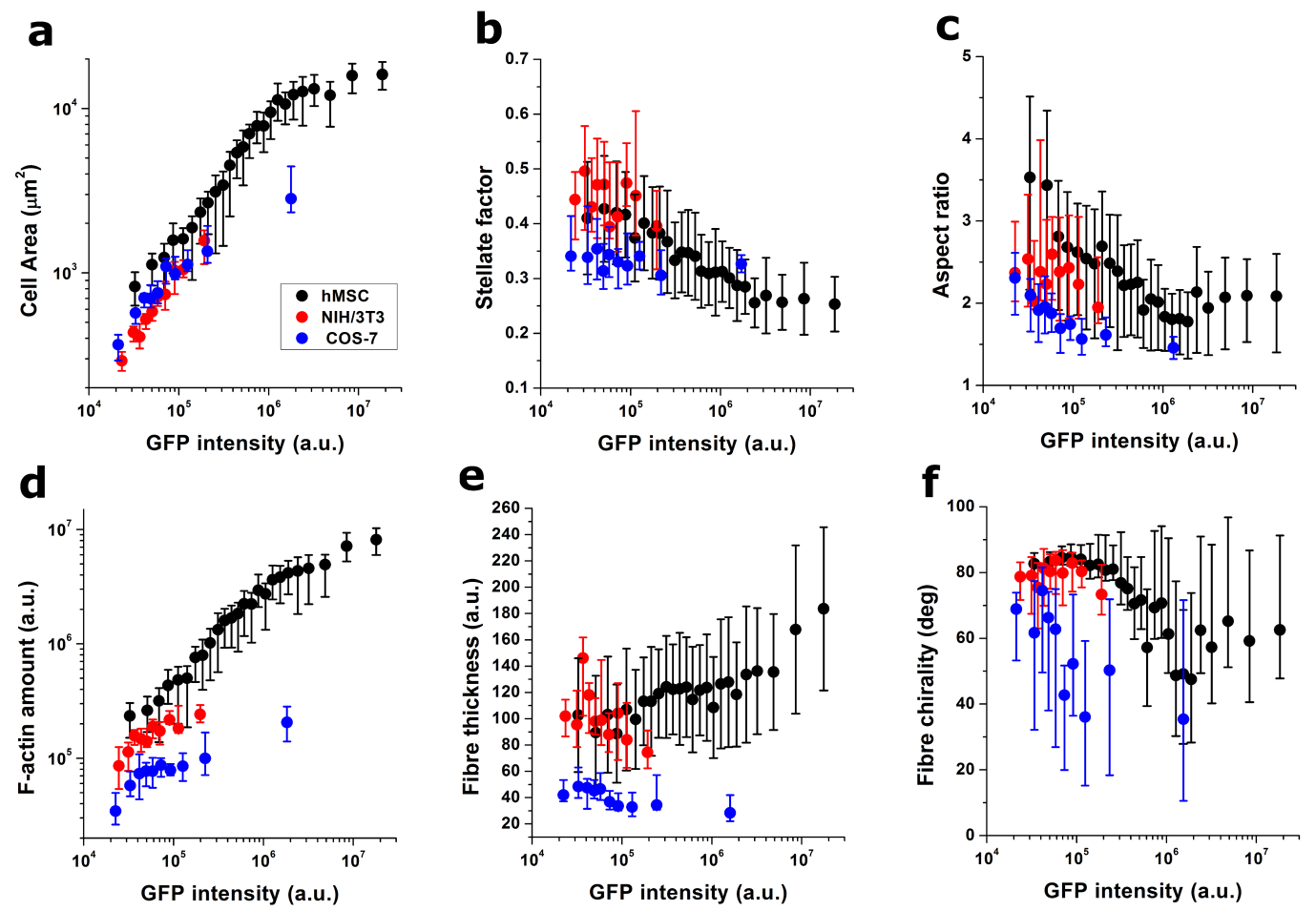
**

**Supplementary Figure 6.** Similar Lifeact-induced effects are observed in several cell types. Dose-response curves quantifying the effect of Lifeact-TagGFP2 expression in human stem cells (black), NIH/3T3 (red) and COS-7 (blue). Plotted is cell spread area (a), cell perimeter stellate factor (b), aspect ratio (c), f-actin amount (d), fibre thickness (d) and chirality of fibres (f). Values for >10 cells were pooled together to compute each individual data point. Data is presented as geometric mean (a and d), mean (b and e) or median (c and f) error bars indicate geometric standard deviation, standard deviation or Q1-Q3, accordingly.


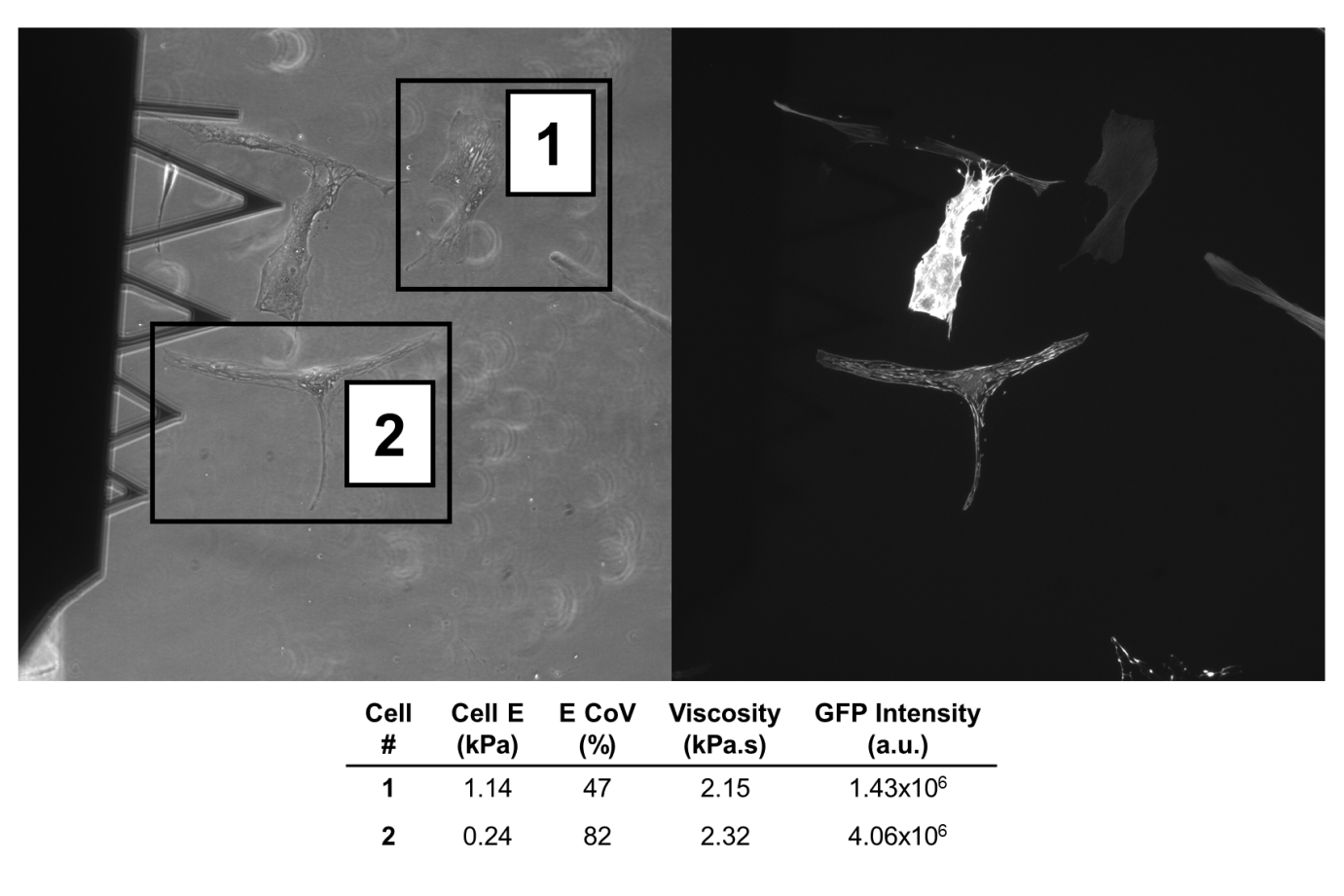


**Supplementary Figure 7** Example of two cells probed using AFM and displaying dissimilar actin organization. Cell #1 displays a nematic-like actin organization and exhibits larger stiffness with reduced CoV, whereas cell #2 displays liquid-like actin bundles and exhibits reduced stiffness with larger CoV. Left panel shows the phase contrast image (including the cantilever chip used for cell probing) and right panel shows the fluorescence image used to quantify Lifeact-TagGFP2 expression. Force-indentation experiments were carried out shortly after these two images were obtained. The whole process of taking the optical images and AFM probing for the two cells lasted < 15 min.

**
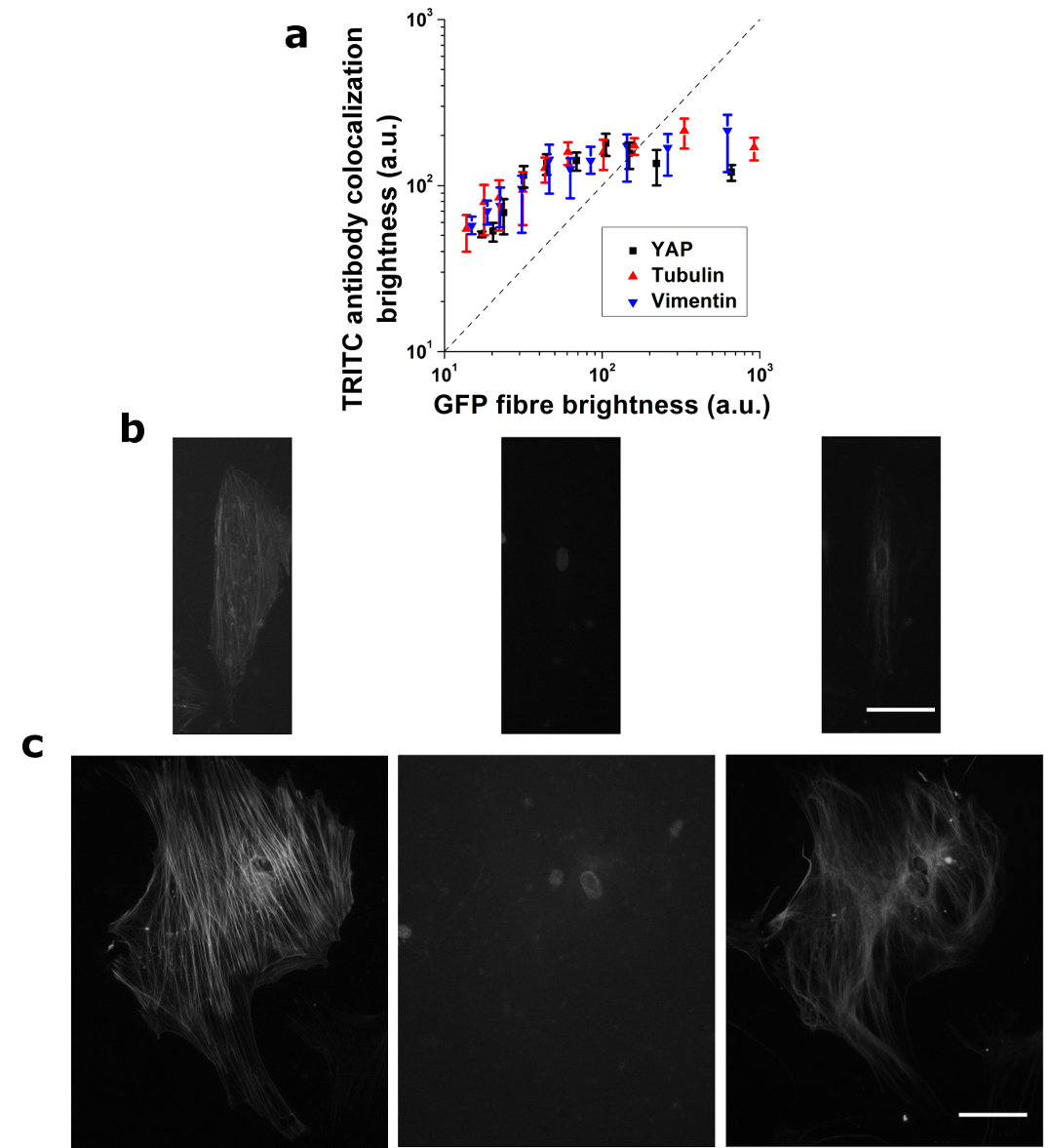
**

**Supplementary Figure 8 Verification of lack of bleed-through between the TagGFP2 and TRITC secondary fluorescence channels.** (a) Relationship between TagGFP2 fibre intensity and colocalized intensities measured on the TRITC channel. All proteins plotted were imaged using the same secondary antibody (goat anti-mouse IgG-TRITC, sc-3796). Note the dashed identity line would correspond to bleed-through behaviour (α=1). Instead, when data was fitted using the function $I_{TRITC}=aI_{TagGFP2}^{\alpha}$, the measured slope α was significantly smaller than 1. YAP, α = 0.156 (-0.041 – 0.354); Tubulin, α = 0.199 (0.078 – 0.320); Vimentin, α = 0.275 (0.188 – 0.362); values in parenthesis correspond to 95% lower and upper confidence levels). (b) Example of a cell displaying no localization of tubulin staining to Lifeact-containing stress fibres, the cell has been transduced with Lifeact (left) subsequently stained with DAPI (middle) and against YAP (right). Scale bar is 20 µm. (c) Example of a cell displaying no localization of vimentin staining to Lifeact-containing stress fibres, the cell has been transduced with Lifeact (left) subsequently stained with DAPI (middle) and against vimentin (right). Scale bar is 20 µm.

**
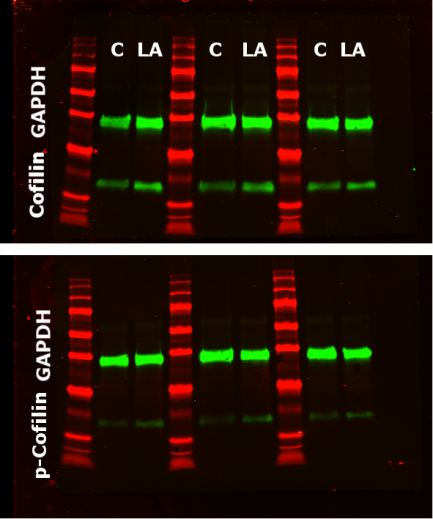
**

**Supplementary Figure 9** Western blots used to quantify cofilin (top gel) and p-cofilin (bottom gel) expression levels relative to GADPH for cell populations treated with control of Lifeact. The pictures has been edited to include labels for blots.

**

**

**Supplementary Figure 10**  Distribution of different cell phenotypes in Lifeact-TagGFP2 transduced samples according to MOI and time of expression. Transduced cells were categorized into three regimes (‘no effect’, dose response and saturation), using the previously identified threshold value for fluorescence expression levels. N>2500 cells.

**Supplementary Video Captions**

**Supplementary Video 1** Time lapse video of migratory behaviour of untrasfected hMSCs. Imaging was conducted over an observation period of 4 days at 1h intervals under controlled environment on a Lumascope 720 system at x10 magnification.

**Supplementary Video 2** Time lapse video of migratory behaviour of hMSCs transfected with Lifeact-TagGFP2 at MOI 250. Imaging was conducted over an observation period of 4 days at 1h intervals under controlled environment on a Lumascope 720 system at x10 magnification.

**Supplementary Video 3** Time lapse video of migratory behaviour of hMSCs transfected with Lifeact-TagGFP2 at MOI 500 Imaging was conducted over an observation period of 4 days at 1h intervals under controlled environment on a Lumascope 720 system at x10 magnification.
